# Characterizing the adult zebrafish model of Parkinson’s disease: a systematic review of dynamic changes in behavior and physiology post-MPTP administration

**DOI:** 10.3389/fnins.2024.1432102

**Published:** 2024-09-10

**Authors:** Khairiah Razali, Jaya Kumar, Wael M. Y. Mohamed

**Affiliations:** ^1^Department of Basic Medical Sciences, Kulliyyah of Medicine, International Islamic University Malaysia (IIUM), Kuantan, Malaysia; ^2^Department of Physiology, Faculty of Medicine, Universiti Kebangsaan Malaysia, Kuala Lumpur, Malaysia; ^3^Clinical Pharmacology Department, Menoufia Medical School, Menoufia University, Shebin El-Kom, Egypt

**Keywords:** Parkinson’s disease, zebrafish, MPTP, intraperitoneal injection, mitochondrial dysfunction

## Abstract

**Introduction:**

Adult zebrafish are increasingly used in Parkinson’s disease (PD) research due to their well-characterized dopaminergic system. Among the toxin-based models, the neurotoxin 1-methyl-4-phenyl-1,2,3,6-tetrahydropyridine (MPTP) is widely utilized to induce parkinsonism in adult zebrafish. Therefore, this review presents an overview of the procedures and the dynamic changes in behavior and physiology observed in the adult zebrafish PD model following a single intraperitoneal injection of MPTP.

**Methods:**

A systematic literature search in the PubMed and Google Scholar databases was conducted to identify relevant articles. Of the 165 articles identified, 9 were included in this review. These chosen articles are original works published before March 2024, all of which utilized adult zebrafish induced with MPTP as the model for PD. Other articles were excluded based on factors such as limited relevance, utilization of zebrafish embryos or larvae instead of adults, and variations in MPTP deliveries.

**Results:**

Studies indicated that the ideal model entails the utilization of mixed gender zebrafish aged between 4 and 6 months from the wild-type strain. The acceptable MPTP doses ranges between 20 μg/g (lowest) and 225 μg/g (highest) and doses above 292 μg/g are lethal. Furthermore, noticeable parkinsonian symptoms appear 1 day after administration and persist for more than 1 week.

**Discussion:**

Mitochondrial dysfunction precedes dopaminergic neurodegeneration within this experimental regime. A single administration of MPTP effectively induces PD in adult zebrafish. This study aids in crafting the adult zebrafish PD model, outlining the progressive behavioral and physiological changes ensuing from MPTP administration.

## 1 Introduction

Neurodegenerative disorders (NDs) pose a rapidly increasing public health concern due to their profound impact on the quality of life of both patients and caregivers. Parkinson’s disease (PD) is the second most common neurodegenerative disorder worldwide, following Alzheimer’s disease, and it primarily affects the elderly population (Mou et al., 2020). Globally, PD is currently impacting around 9.4 million individuals, with projections estimating an increase to 12 million by 2040 (Ou et al., 2021). Parkinson’s disease is characterized as a neurological condition predominantly marked by motor impairments, including resting tremors, bradykinesia, and postural instability (Jankovic and Tan, 2020). As the disease progresses into later stages, it can also give rise to non-motor symptoms, including cognitive impairments, sleep disturbances, gastrointestinal dysfunction, and depression (Kouli et al., 2018).

The main pathological features of PD involve the significant loss of nigral dopaminergic neurons and its dopamine projection to the striatum via the nigrostriatal dopaminergic pathway, as well as the formation of Lewy bodies (LBs; Kouli et al., 2018; Zhou et al., 2023). These neurons and their dopamine projections are vital for regulating and modulating voluntary motor movements, posture, and coordination. Reportedly, nigrostriatal pathway has consistently been identified as the most severely damaged in PD (Caminiti et al., 2017). While the etiology of PD is predominantly sporadic, the loss of dopaminergic neurons in the substantia nigra is thought to be the result of a complex interplay among various factors, including mitochondrial dysfunction, oxidative stress, inflammation, and inadequate protein degradation (Chang and Chen, 2020; Isik et al., 2023). Nonetheless, significant questions remain concerning the exact causes and mechanisms behind the specific death of these neurons in PD.

To address these questions, various animal models of PD have been developed, with a particular focus on elucidating the early or dynamic neurodegenerative events. This is of great significance since most PD cases in humans are usually diagnosed in the advanced stages of the disease when opportunities for prevention and cure are already limited. Two animal models commonly used in studying neurodegenerative diseases are toxin-induced and transgenic models (Banerjee et al., 2022). When it comes to PD research, the most prevalent method of developing animal models involves the use of a neurotoxin known as 1-methyl-4-phenyl-1,2,3,6-tetrahydropyridine (MPTP). Intraperitoneal (i/p) administration of this neurotoxin to animal models can recapitulate several, albeit not all (most studies reportedly failed to mimic LBs formation), significant key features of PD, including the loss of midbrain dopaminergic neurons, depletion of dopamine in the striatum, and behavioral deficits (Meredith and Rademacher, 2011; Mustapha and Taib, 2021).

While MPTP mouse models are currently the predominant choice for PD studies, the MPTP zebrafish model is gaining increasing recognition and attention in the scientific community. With respect to genetic similarity, zebrafish share 71.4 percent of the same genes with humans (Howe et al., 2013). Moreover, zebrafish are known for their higher fecundity, ease of handling, and relatively shorter time to reach adulthood when compared to primates and rodents (Adhish and Manjubala, 2023). Studies involving the use of MPTP for developing PD model in zebrafish have yielded promising results, particularly in mimicking movement disorders and molecular dysfunction akin to those seen in PD. Nevertheless, most of these studies have favored the use of zebrafish embryos and larvae over adult zebrafish, likely due to their abundance and ease of handling. In cases where research goals pertain to mature brain functions or when aging plays a role, adult zebrafish provide a more accurate platform for observation, as the brains of zebrafish embryos and larvae are still in the developmental stage.

Therefore, to further encourage the use of adult zebrafish model for PD research, we present a comprehensive review of the protocols used to induce MPTP in adult zebrafish, encompassing details on zebrafish selection, as well as dosages, administration frequency, and i/p method of MPTP delivery. In addition, this review underscores the dynamic changes within the nigrostriatal pathway of MPTP-induced zebrafish, as documented in published studies. To systematically structure this review, the PICO framework was adapted, where: Population - adult zebrafish model of PD; Intervention - single i/p injection of MPTP; Comparative intervention - untreated or vehicle-treated adult zebrafish; and Outcomes: MPTP toxicodynamics in adult zebrafish.

## 2 Materials and methods

### 2.1 Search strategy

A systematic review of published literature on the effects of single MPTP injection on adult zebrafish PD model was performed in accordance with the PRISMA (Preferred Reporting Items for Systematic reviews and Meta-Analyses) protocol.

A comprehensive search of the literature published until 5 March 2024 was conducted in PubMed database (US National Library of Medicine, USA) using the terms “zebrafish” and “MPTP” as well as Google Scholar database with the following search prompt: “adult zebrafish” AND “MPTP” AND “intraperitoneal” NOT “larva” NOT “larvae” NOT “embryo” NOT “embryos.” The Harzing’s Publish or Perish software (Windows GUI Edition, Tarma Software Research Ltd., UK) was used to aid the data collection process.

### 2.2 Eligibility criteria

Included in this review were studies that: (i) utilized adult zebrafish as the model; (ii) performed one-time administration of MPTP via i/p injection, regardless of the injection dose or the timing of assessment; and (iii) original research. Articles excluded from the final selection were: (i) those using embryos or larvae instead of adult zebrafish; (ii) those administering MPTP through routes other than i/p injection; (iii) those administering drugs other than MPTP; (iv) those categorized as review papers; (v) non-English articles; and (vi) no full text available.

### 2.3 Selection process, data preparation, and extraction

Initially, all articles retrieved from the databases were pooled together in a single Excel sheet and duplicates were removed. Then, the remaining articles were screened and selected by reading the titles and abstracts, followed by careful reading of the full texts. Whenever full-text articles were unavailable, requests for them were made by emailing the corresponding authors. Articles that did not meet the inclusion criteria were excluded. The article selection was conducted independently and final agreement between the authors was reached.

To ensure consistency and facilitate comparisons, this review presented the same parameter using uniform units, even if a specific article originally used different units. For instance, MPTP dosages were standardized and reported as μg/g across all articles.

For each selected article, the following data items were extracted: characteristics of adult zebrafish model (strain, age, gender, body weight, number of fish per group), MPTP dose, time of behavior and molecular assessments, as well as the findings. To stick to the aim of this review, only the findings that are compatible with the inclusion criteria are reported and discussed. The behavioral and molecular findings are discussed in the next sections to elaborate the toxicodynamics of MPTP on adult zebrafish.

## 3 Results

### 3.1 Selection of studies

**Figure 1:**
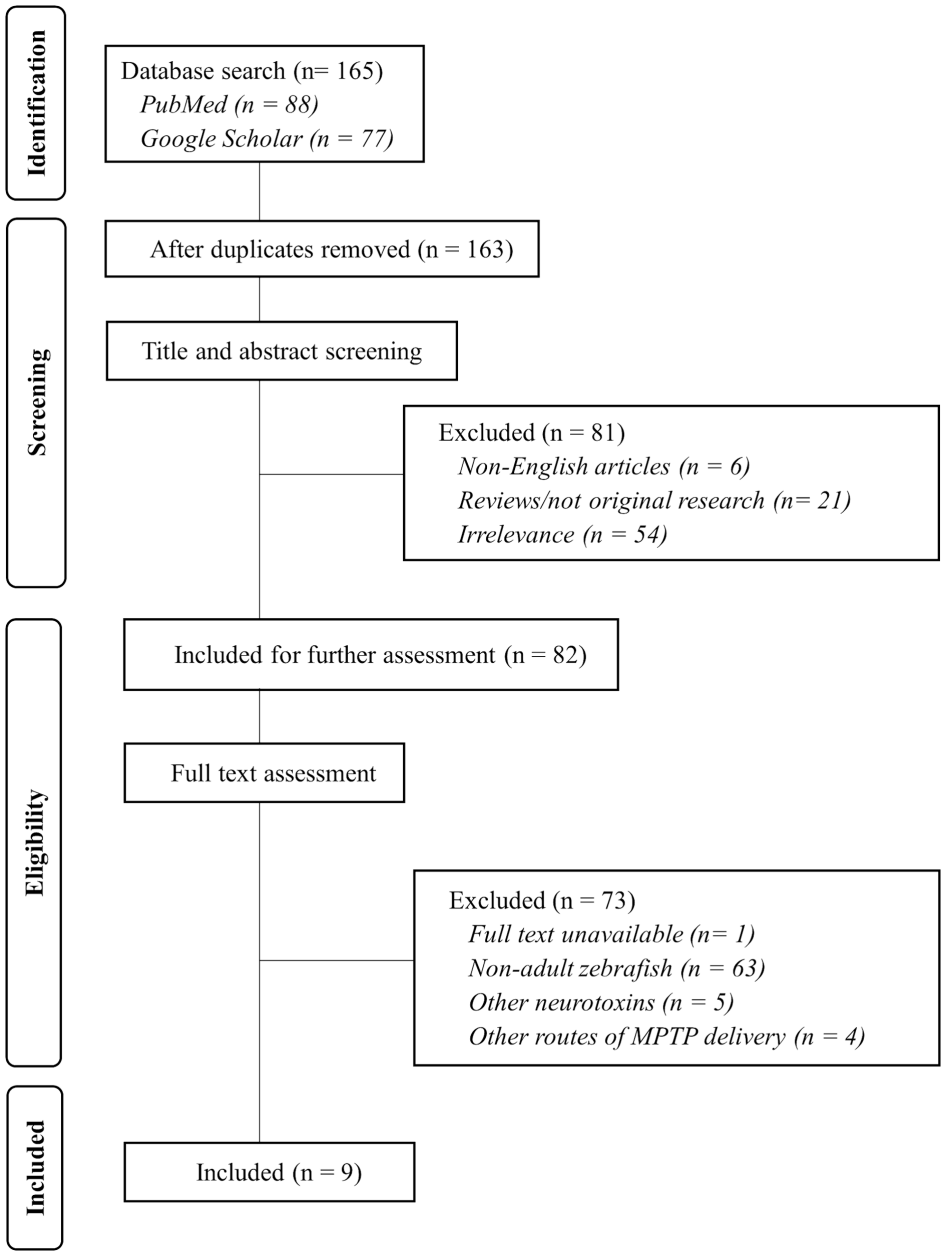
Flow diagram of the study selection process. Nine articles were selected to be further evaluated in this study. Flow diagram adapted from PRISMA 2020 Reporting Guidelines.

The selection process is visualized in Figure 1, which provides a flowchart outlining the steps taken in the selection of articles for this review. An initial total of 165 articles were retrieved from PubMed and Google Scholar databases. After removing duplicates and screening the titles and abstracts, 81 articles were excluded based on the listed exclusion criteria (non-English, not original research, and irrelevance). Then, through full text assessment, a further 73 articles were excluded due to no available full text as well as utilization of zebrafish other than adults, neurotoxins other than MPTP, and injections other than i/p.

Finally, nine articles were identified that met the criteria and effectively administered MPTP through a single i/p injection to create a PD model in adult zebrafish. In this review, our emphasis was on a specific regimen involving a single i/p injection of the MPTP neurotoxin. Although we recognize that there are studies that have utilized multiple MPTP injections and alternative routes of administration, such as intramuscular, cerebroventricular, and immersion, we chose the most prevalent regimen (a single i/p injection) to offer a more comprehensive and in-depth analysis of the research findings.

### 3.2 Characteristics of the included studies

The adult zebrafish model used by these studies were mostly of the wild type (WT) strain of age ranging between 4 and 6 months old. The MPTP dose administered varied across studies, with 20 and 225 μg/g being the smallest and highest dose, respectively. In addition, most of these studies examined the early effects of MPTP shortly after injection (specifically, 1 day post-injection), and three of them additionally explored the later effects of MPTP (beyond 5 days post-injection). The specifics of the nine selected articles are summarized in Table 1.

**Table 1:** Detailed experimental variables of the selected articles, including zebrafish selection, MPTP doses, and assessment timing.

Authors	Adult zebrafish model					MPTP paradigm				Assessments	
	Strain	Age	Gender	Body weight (g)	Fish per group	Dose (μg/g)	Method of i/p injection	Injection volume (μl/g)	Solvent	Time	Type
Bretaud et al. (2004)	AB and EK	N/A	N/A	0.3	5	225	N/A	5	Saline	0 – 6^th^ day post-injection	Locomotor activity; Physical appearances
Nellore et al. (2013)	WT	< 8 months	N/A	N/A	6–8	225	Hamilton syringe	5	Saline	5^th^ day post-injection	Locomotor activity; Enzyme analyses
Sarath Babu et al. (2016)	WT	6 months	Mixed	N/A	10	50	N/A	5	Sterile water	1^st^ day post-injection	Locomotor activity; Protein and gene analyses
Selvaraj et al. (2019)	WT	5 months	N/A	N/A	N/A	100	Insulin syringe	20	Saline	1^st^ day post-injection	Locomotor activity; HPLC analysis; Gene analysis
Bashirzade et al. (2022)	WT	~ 1 year	Mixed	1.0	9–19	200	N/A	100	DMSO	1^st^ day post-injection	Locomotor activity; Cognitive behaviors
Razali et al. (2022)	WT	3–4 months	Mixed	0.8–1.0	20	200	31G needle	30	Saline	0, 1^st^, and 4^th^ day post-injection	Locomotor activity; Body weight analysis
Razali et al. (2023)	WT	~ 5 months	Mixed	0.8–1.0	10	20	31G needle	40	Saline	1^st^ and 2^nd^ day post-injection	Locomotor activity; Gene analysis
Omar et al. (2023a)	N/A	4–6 months	N/A	N/A	6	100	30G needle	10	Saline	1^st^, 3^rd^, 5^th^, 10^th^, and 30^th^ day post-injection	Locomotor activity; Gene analysis; Protein analysis; IHC assay
Omar et al. (2023b)	WT	4–6 months	N/A	N/A	6–10	100	30G needle	10	Saline	3^rd^, 5^th,^ and 10^th^ day post-injection	Locomotor activity; Gene analysis; IHC and ELISA assays

### 3.3 Selection of the zebrafish

The selection of an appropriate animal model for research is essential to ensure scientific validity, practical feasibility, and replicability of research outcomes. Employing a well-established and widely used animal model can enhance the consistency and comparability of the outcomes (Eggel and Würbel, 2021). Besides, opting for an animal model that closely mirrors relevant aspects of human biology, such as utilizing adult zebrafish as a representation of human adulthood, heightens the probability of generating findings that are transferable to human biology.

**Figure 2:**
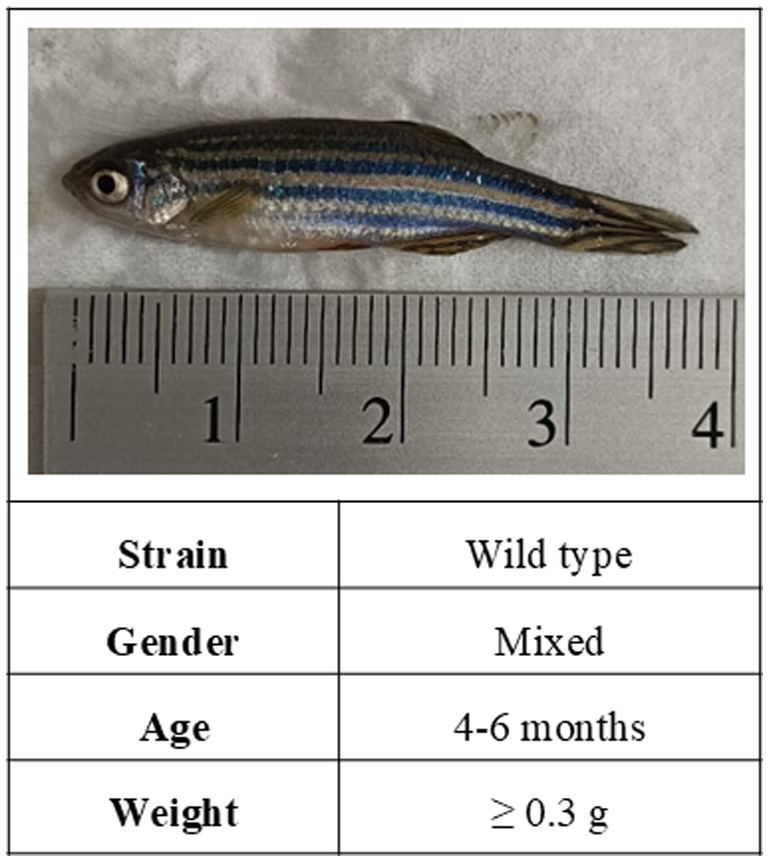
The characteristics of the adult zebrafish model commonly used in MPTP-induced PD studies.

In mice research, Jackson-Lewis and Przedborski (Jackson-Lewis and Przedborski, 2007) have documented that gender, age, and body weight are critical factors affecting the sensitivity and consistency of MPTP lesions. Similarly, when it comes to zebrafish research, the choice of zebrafish model may impact the toxicity effects of MPTP. While studies involving MPTP-induced adult zebrafish are not as abundant as those in mice, our review of the selected articles indicates that the most utilized adult zebrafish PD model for investigating MPTP neurotoxicity is the wild-type (WT) strain, comprising mixed-gender zebrafish aged between 4 and 6 months in average, and weighing at least 0.3 g (Figure 2).

The zebrafish WT strain represents a group of zebrafish that have not undergone selective breeding or genetic modification. This strain is most typically used in behavioral tests (Audira et al., 2020). The wild-type strain, characterized by its greater genetic diversity, offers a better representation of the genetic variation observed in the human population. In PD research, given that PD is a sporadic and idiopathic disease that can affect any individual in a population, the use of the wild-type strain serves to mimic this genetic heterogeneity effectively. Notably, Bretaud et al. (2004) detailed the use of AB and EK strains in their study. It is worth acknowledging that the term “wild type” encompasses various strains, including AB and EK strains (Audira et al., 2020; Dahlén et al., 2019).

Parkinson’s disease affects men and women differently. While men are at twice the risk of developing PD compared to women, women experience higher mortality rates and faster disease progression (Cerri et al., 2019). In mouse models, male PD mice have been preferred for their hormonal stability, leading to more consistent results. Additionally, certain administration protocols and assessments have revealed that female mice exhibit heightened sensitivity to MPTP toxicity (Antzoulatos et al., 2011; Torres-Rojas and Jones, 2018). Nevertheless, recent research suggested that these differences may be more attributable to individual variability rather than inherent gender differences (Becker et al., 2016; Lovick and Zangrossi 2021). In our review of selected articles, half incorporated both male and female zebrafish, while the other half did not specify gender. To the best of our knowledge, there is no available research that compares gender-related differences in MPTP susceptibility in zebrafish. While mixed-gender studies better reflect human diversity, exploring sex-related differences in MPTP-induced zebrafish remains an intriguing area for investigation.

When selecting the age of animals for a PD model, several crucial factors must be considered. Given that PD predominantly affects older individuals, using older zebrafish is more suitable for replicating the typical age of PD onset in humans, especially concerning behavioral changes (motor and non-motor symptoms) and susceptibility to neurodegeneration. Ideally, adult zebrafish should be at least 3 months old, considering that zebrafish typically reach adulthood by this age. As outlined in Figure 2, the typical age range for modeling PD in adult zebrafish falls between 4 and 6 months. Furthermore, it is crucial to factor in cost and time constraints. Maintaining one-year-old zebrafish can be more expensive and time-consuming compared to its three-month-old counterparts. Hence, taking these factors into account, the age range of 4 and 6 months is indeed the most suitable and optimal age for inducing MPTP in adult zebrafish for PD modeling.

### 3.4 Intraperitoneal injection of MPTP

A frequently used approach for drug delivery in animal research involves i/p injection, a process in which a needle is used to inject the drug into the peritoneal cavity. In zebrafish, this peritoneal cavity is located within the abdominal cavity and is lined by a serous membrane called the peritoneum (Kinkel et al., 2010). Due to the relatively small size of zebrafish, i/p injections are typically conducted using a microinjector or manually with a syringe equipped with a very fine needle.

From our review of the selected articles, in terms of the injection technique, the Hamilton syringe and insulin syringe equipped with a 30G or 31G needle are the most frequently employed tools. It is advisable to steer clear of larger needles (29G and below) as they are too large for zebrafish and carry an increased risk of causing harm to the fish. In terms of the dosages, the documented MPTP doses for this regimen vary, with the lowest being 20 μg/g body weight (hereinafter referred to as μg/g) and the highest reaching 225 μg/g. According to research by Bashirzade et al. (2022), the lethal dose (LD¬50) for MPTP exposure through i/p injection in adult zebrafish is estimated to be 292 μg/g. When utilizing MPTP to induce PD in animal models, it is vital to avoid the lethal dose to prevent false-positive results. In such cases, the observed pathology or death in animals may result from acute toxicity rather than accurately reflecting PD progression. Consequently, an MPTP dose exceeding 292 μg/g is not recommended for adult zebrafish. However, it is important to emphasize that the lethal dose is influenced by factors such as the strains, sizes, and individual sensitivity of the animals, as well as the methods of drug administration. Thus, each research study is advised to conduct its own LD50 toxicity assay to ascertain the lethal dose specific to their animals.

**Figure 3:**
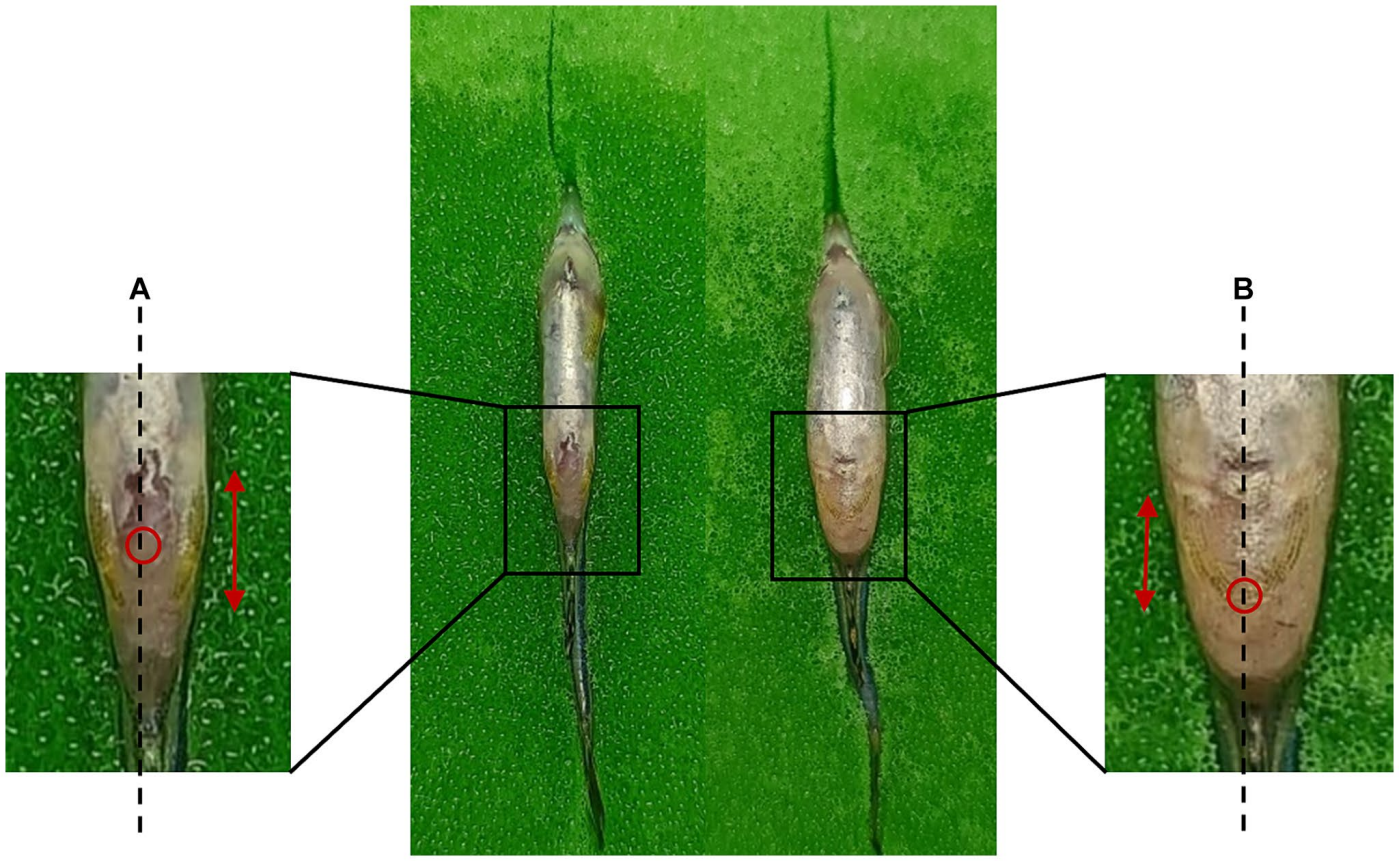
Intraperitoneal injection sites for male and female zebrafish. **(A)** Male: the injection site is located midway along the length of the pelvic fin. **(B)** Female: the injection site is positioned at the base of the pelvic fins. Dashed line: midline; Double-headed arrow: pelvic fin length; Red circle: injection site.

For precise targeting of the peritoneal cavity, one approach involves injecting at a point located posterior to the pelvic girdle, specifically at the midline between the pelvic fins (Omar et al., 2023a; Kinkel et al., 2010; Samaee et al., 2017) and inserting the needle to a depth of around 1 to 2 mm (Razali et al., 2022; Ma et al., 2018). Figure 3 provides a visual representation of the injection site. Alternatively, the peritoneal cavity can be accessed from the side of the zebrafish by inserting the needle to a depth of 3 to 4 mm (Ma et al., 2018). Given the limited capacity of the peritoneal cavity, it is advisable to administer a maximum of 50 μl of the drug at one time. Additionally, it is common practice to withhold food from the zebrafish for 24 h before injection to create more space between the peritoneal cavity and the abdominal organs (Omar et al., 2023a).

i/p injection remains the preferred method of MPTP delivery into adult zebrafish, it is important to acknowledge the use of alternative routes, including intramuscular (i/m) injection (Anichtchik et al., 2004) and cerebroventricular microinjection (CVMI; Kalyn and Ekker, 2021). The former involves inserting needles at a 45° angle relative to the spine and anterolateral to the dorsal muscles (Rihel and Ghosh, 2016), while the latter involves employing a microinjector in the cerebroventricular region of the brain (Kizil and Brand, 2011). Comparing these three methods, i/p injection is relatively simpler and has lower associated risks. Improper execution of i/m injection carries the risk of nerve damage (Borst et al., 2013), a concern mitigated by the abdominal targeting of i/p injection. Moreover, the challenges posed by the small size of the zebrafish make CVMI a less straightforward method, requiring greater expertise and the use of sophisticated instruments. Despite these considerations, the selection of the MPTP delivery method depends on the specific objectives and experimental conditions of each study.

In a summary, the most used method for inducing PD in the adult zebrafish model is through an injection of not more than 292 μg of MPTP per gram of zebrafish body weight using a 30G (or 31G) needle attached to either a Hamilton or insulin syringe. As an additional point, it is worth noting that while i/p injection is generally a safe method, it is advisable to incorporate a recovery period following the injection to closely monitor for any injection-related complications, including issues like bleeding or hyperventilation.

### 3.5 MPTP toxicokinetics on adult zebrafish

Regarding the practical use of MPTP, our review revealed that seven out of nine articles employed MPTP purchased from Sigma Aldrich, USA (Cat# M0896). This MPTP is conjugated with hydrochloride (C12H15N·HCl) and has a molecular weight of 209.72. As advised by Jackson-Lewis and Przedborski (Jackson-Lewis and Przedborski, 2007), it is essential to account for the presence of HCl when preparing the injection solution to ensure the accurate amount of MPTP per dose. Detailed calculations for this process can be found in their published protocols (Jackson-Lewis and Przedborski, 2007). In the context of MPTP solubility, it is commonly dissolved in either a normal saline solution or a dimethyl sulfoxide (DMSO) solution. The presence of sodium chloride in the normal saline and the organic properties of DMSO aid in improving the solubility and stability of MPTP in an aqueous solution. Among the nine articles we reviewed, seven utilized normal saline as the solvent to dissolve MPTP, while the remaining two articles used DMSO and sterile water as their respective solvents.

Initially synthesized in 1947 and then reappearing in the 1980s, MPTP (C12H15N) came to the forefront of scientific attention when seven young adults experienced parkinsonism after self-injecting a synthetic heroin (Langston, 2017; Nonnekes et al., 2018). Investigation into this heroin, led by Dr. Langston, unveiled that it consisted of almost pure MPTP compound, a neurotoxin that specifically targets dopaminergic neurons in the SNpc (Langston, 2017). In the following decades, MPTP became the most widely used neurotoxin for developing toxin-induced PD models in animal research, significantly contributing to our understanding of PD pathological mechanisms. MPTP is highly lipophilic, which enables it to easily penetrate the lipid bilayer membranes and thus, freely crosses the blood–brain barrier (BBB). This characteristic of MPTP allows it to have rapid toxicokinetics.

When successfully administered intraperitoneally in adult zebrafish, MPTP in the peritoneal cavity is absorbed into the bloodstream. The peritoneal cavity has an extensive network of capillaries that facilitate absorption (Al Shoyaib et al., 2020). Once entered, MPTP travels through systemic circulation to reach the central nervous system (CNS). Due to its lipophilicity, MPTP easily crosses the BBB and enters the brain. Nonetheless, it is important to note that not 100 percent of the administered MPTP will enter the brain; some may circulate and reach other organs, be metabolized, or undergo excretion from the body. Therefore, the actual amount of MPTP that exerts its effects on the zebrafish brain may be less than the total dose administered.

Within the brain, MPTP is metabolized by monoamine oxidase B (MAO-B), which is predominantly secreted by glial cells, particularly astrocytes. MAO-B catalyzes the conversion of MPTP into an intermediate metabolite known as 1-methyl-4-phenyl-2,3-dihydropyridine (MPDP+), which subsequently proceeds to form the toxic metabolite 1-methyl-4-phenylpyridinium (MPP+; Mustapha and Taib, 2021). Evaluating MAO-B activity within the brain is a validated method for confirming the successful delivery of MPTP to the brain (Jackson-Lewis and Przedborski, 2007). Reportedly, zebrafish possess a single paralogue of the monoamine oxidase enzyme (also known as zMAO), in contrast to the two isoforms found in humans (MAO-A and MAO-B; Fierro et al., 2013). Studies indicated that while zMAO is structurally and functionally more like human MAO-A rather than MAO-B, it is still susceptible to inhibition by deprenyl, a compound recognized as a selective inhibitor of MAO-B (Aldeco et al., 2011; Horzmann and Freeman, 2016). Additionally, the administration of deprenyl in adult zebrafish mitigated MPTP-induced impairments (Sarath Babu et al., 2016). This finding suggests that although zMAO is more analogous to human MAO-A, it may serve a role comparable to MAO-B in the metabolism of MPTP. Further research on zMAO is required to gain a deeper understanding of its involvement in the pharmacokinetics of MPTP.

**Figure 4:**
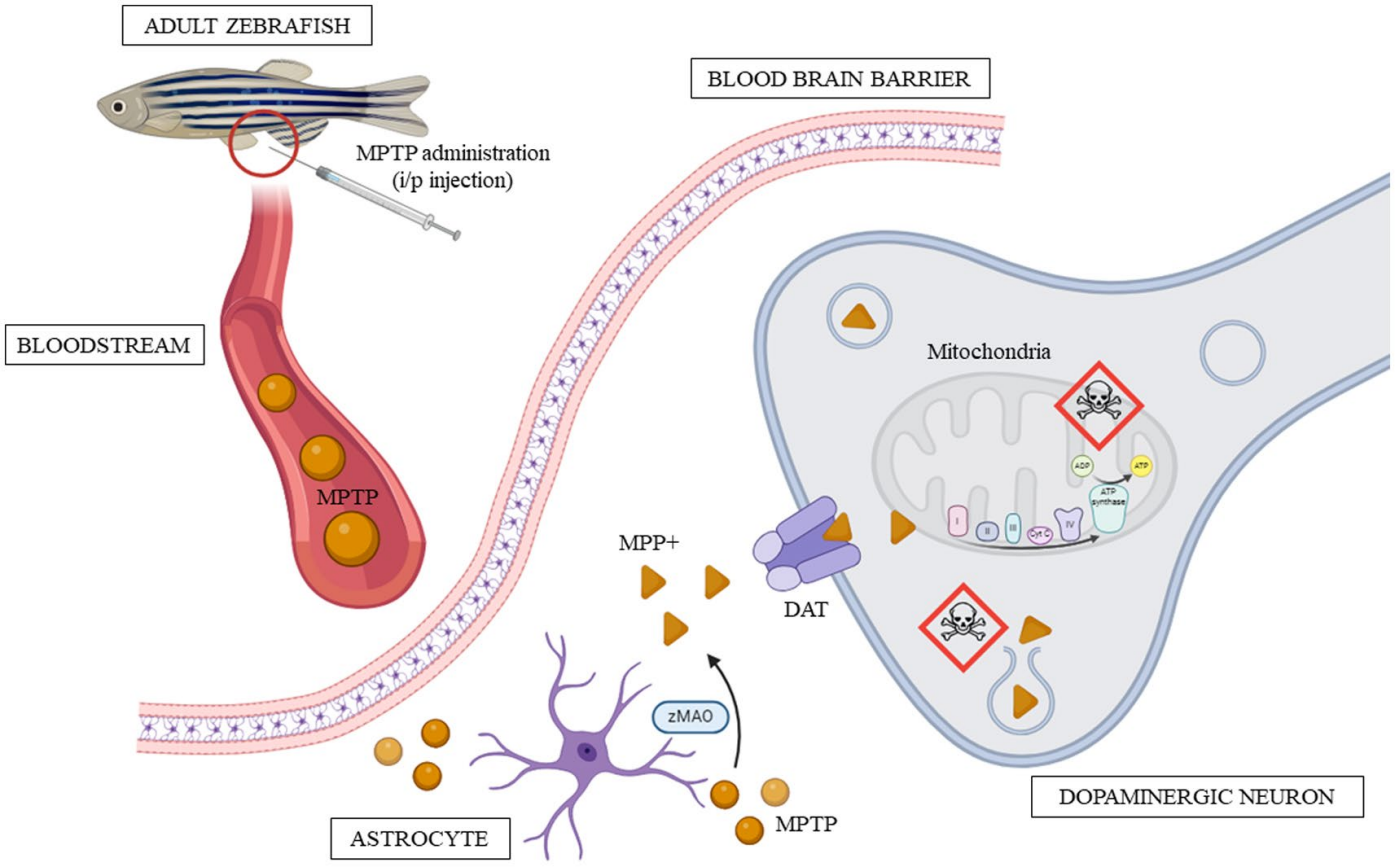
Toxicokinetics of MPTP in adult zebrafish from the point of administration to the onset of toxicity. The red circle marks the site of intraperitoneal injection, while the skull icon indicates where the toxin mainly takes effect.

Due to its structural resemblance to dopamine, MPP+ enters dopaminergic neurons selectively by utilizing the dopamine transporter (DAT). In zebrafish, the DAT is encoded by the slc6a3 gene, which is orthologous to the human DAT (Wang et al., 2019). Apart from DAT, MPP+ also exhibits a high affinity for norepinephrine and serotonin transporters (Mustapha and Taib, 2021). Once inside dopaminergic neurons, MPP+ accumulates within the mitochondria and is also stored within synaptosomal vesicles (Mustapha and Taib, 2021). Within the mitochondria, MPP+ disrupts the activity of complex I in the mitochondrial electron transport chain (ETC). This disruption hampers the normal flow of electrons and the generation of ATP, resulting in energy depletion and mitochondrial dysfunction (Meredith and Rademacher, 2011). Simultaneously, MPP+ sequestration within synaptosomal vesicles can lead to neuronal damage when it surpasses a certain accumulation threshold (Mustapha and Taib, 2021). This disturbance in dopaminergic neuron homeostasis, primarily affecting mitochondrial function, triggers a series of cascading events, including oxidative stress and inflammation, ultimately resulting in neuronal death (Huang et al., 2017). Notably, mitochondrial dysfunction has gained increasing attention as a contributing factor to the etiology of PD (Chen et al., 2019; Gao et al., 2022; Grünewald et al., 2019). Figure 4 describes the toxicokinetics of MPTP after it is intraperitoneally injected into adult zebrafish.

Taken together, this comprehensive overview highlights the toxicokinetics of MPTP on humans and zebrafish biology, emphasizing the need for continued research to deepen our understanding of its mechanisms and implications in PD.

### 3.6 MPTP toxicodynamics on adult zebrafish

In this section, we consolidate and analyze findings from the selected studies with the aim of comprehending the toxicodynamics of MPTP on adult zebrafish, focusing on the immediate, early, and late effects, both behaviorally and at the molecular level. Modified from Huang et al. (2017), we categorize immediate effects as those observed within 24 h post-injection, early effects as those occurring during the first week, and late effects as those manifesting after 1 week or more following MPTP injection.

#### 3.6.1 Immediate, early, and late MPTP effects on adult zebrafish behavior

When studying zebrafish locomotion, swimming behavior tests are commonly used to examine specific parameters reflecting their locomotor activity (Li et al., 2018; Tan et al., 2022). These typically include total distance, mean speed, and the number of crosses. Total distance refers to the overall distance covered by the zebrafish during the test period, usually measured in cm or mm. Our review indicates that under normal conditions, adult zebrafish can travel approximately 500–700 cm within 3 min. However, this distance can vary based on the tank size and water volume used in the assay. Mean speed represents the average distance swum per second, often measured in cm/s or mm/s. Typically, adult zebrafish can swim up to 6 cm/s. The number of crosses indicates how frequently the zebrafish navigate back and forth within designated zones in the assay tank. A higher crossing frequency suggests greater mobility. It is essential to acknowledge that behavioral analysis can be influenced by individual differences. Some zebrafish might exhibit higher anxiety levels compared to others in the same group and batch, potentially resulting in performance outliers. The experimental setup used for the behavioral assessment in each study is presented in Table 2.

**Table 2:** Detailed setup parameters for behavioral assessment conducted in the selected studies.

Authors	Setup parameters			
	Tank dimensions (cm)	Habituation duration	Test duration	Zone divisions
Bretaud et al. (2004)	25 (L) × 16.5 (W) × 6.5 (H)	N/A	5 min	4 zones
Nellore et al. (2013)	30 (L) × 10 (W) × 15 (H)	10 min	5 min	4 zones
Sarath Babu et al. (2016)	N/A	N/A	2 min	2 zones
Selvaraj et al. (2019)	22.5 (L) × 15.5 (W) × 14 (H)	5 min	3 min	N/A
Bashirzade et al. (2022)	25 (L) × 8 (W) × 15 (H)	N/A	10 min	Y-shaped tank
Razali et al. (2022)	24 (L) × 13.5 (W) × 13.5 (H)	5–10 min	3 min	2 zones
Razali et al. (2023)	24 (L) × 13.5 (W) × 13.5 (H)	5 min	3 min	4 zones
Omar et al. (2023a)	N/A	5 min	5 min	2 zones
Omar et al. (2023b)	N/A	N/A	5 min	2 zones

Following an acute MPTP administration, there were no immediate changes observed in the swimming parameters of adult zebrafish. Behavioral assessments conducted on Day 0 (Razali et al., 2022; Razali et al., 2023) revealed no statistically significant differences in total distance and average speed. Correspondingly, Bretaud et al. (2004) documented insignificant differences in the number of crosses between MPTP-induced zebrafish and the control group. This indicates that, at least in this specific context and dosage (Bretaud et al., 2004: 225 μg/g; Razali et al., 2022: 200 μg/g; Razali et al., 2023: 20 μg/g), the immediate locomotor impact of MPTP is negligible. Despite the rapid toxicokinetics of MPTP, it did not visibly impact the locomotion of zebrafish immediately.

The early effect of MPTP on the locomotor activity of adult zebrafish became noticeable 1 day after administration (Table 3). During the behavioral test on Day 1 following MPTP administration, there was a notable reduction in total distance, mean speed, and the number of crosses. Analysis of the collected data (Bretaud et al., 2004; Sarath Babu et al., 2016; Selvaraj et al., 2019; Razali et al., 2022; Razali et al., 2023; Omar et al., 2023a; Omar et al., 2023b) revealed that MPTP-induced zebrafish traveled less distance and swam at slower speeds compared to the control group. Additionally, they displayed reduced movement frequencies across different zones (Sarath Babu et al., 2016; Razali et al., 2023). Nevertheless, Bretaud et al. (2004) and Bashirzade et al. (2022) did not report similar outcomes. Bretaud et al. (2004) found that noticeable decreases in locomotor activity were only seen beginning on the second day after administration. As anticipated, the decrease continued Day 3, 5, and 6 after treatment (Bretaud et al., 2004), in agreement with other studies. Although there were no discernible disparities in locomotion, Bashirzade et al. (2022) reported a decline in cognitive phenotypes through AI-based platform analysis. The utilization of AI-based analysis holds promise for future endeavors as it has the potential to enhance sensitivity and reliability of behavioral analyses.

**Table 3:** The immediate, early, and late effects of MPTP on the locomotor activity of adult zebrafish, characterized by swimming parameters.

Swimming parameters	Immediate effects	Early effects						Late effects		References
	Day 0	Day 1	Day 2	Day 3	Day 4	Day 5	Day 6	Day 10	Day 30	
Total distance										Bretaud et al. (2004)
						↓↓				Nellore et al. (2013)
		↓								Sarath Babu et al. (2016)
		↓↓↓								Selvaraj et al. (2019)
		ns								Bashirzade et al. (2022)
	ns	↓↓			↓↓					Razali et al. (2022)
	ns	↓↓	↓↓↓							Razali et al. (2023)
		↓	↓↓	↓↓		↓↓		↓↓	ns	Omar et al. (2023a)
				↓		↓		↓		Omar et al. (2023b)
Average speed										Bretaud et al. (2004)
						↓↓↓				Nellore et al. (2013)
										Sarath Babu et al. (2016)
		↓↓								Selvaraj et al. (2019)
		ns								Bashirzade et al. (2022)
	ns	↓↓			↓↓					Razali et al. (2022)
	ns	↓↓	↓↓↓							Razali et al. (2023)
		↓	↓↓	↓↓		↓↓		↓↓	ns	Omar et al. (2023a)
				↓		↓		↓		Omar et al. (2023b)
No. of crosses	ns	ns	↓↓	↓↓	↓	↓↓	↓↓			Bretaud et al. (2004)
						↓↓				Nellore et al. (2013)
		↓↓								Sarath Babu et al. (2016)
										Selvaraj et al. (2019)
										Bashirzade et al. (2022)
										Razali et al. (2022)
	ns	↓↓	↓↓↓							Razali et al. (2023)
										Omar et al. (2023a)
										Omar et al. (2023b)

*Empty brackets: no data available. *↓: 0–25% reduction, ↓↓: 26–50% reduction, ↓↓↓: 51–75% reduction, ↓↓↓↓: 76–100% reduction, ns: no significant changes.

Notably, the significant changes in swimming parameters persisted up to the sixth day (Table 3). Hence, it can be inferred that MPTP seems to have an early and sustained impact on the locomotor activity of zebrafish, with the effects becoming more pronounced in the days following administration, rather than immediately.

The noticeable reductions in total distance and average speed persist even on the 10th day of post-MPTP administration (Omar et al., 2023a; Omar et al., 2023b). Nonetheless, after 30 days, the late effects of MPTP reversed, showing a recovery process that restored the swimming parameters to their normal levels (Omar et al., 2023a). These findings align with the recognized transient effects of MPTP observed in other animal studies, signifying a common trend of reversible effect on locomotor activity following MPTP exposure. Additionally, a separate study involving cerebroventricular microinjection (CVMI) of MPTP in adult zebrafish corroborates this pattern. This study suggests that the impact of MPTP administered through CVMI is transient, lasting up to approximately 2 weeks (Kalyn and Ekker, 2021).

Taken together, the documented data suggest that a single administration of MPTP with dose ranging from 20 to 225 μg/g, induces significant locomotor impairments within 24 h after administration in adult zebrafish, and these impairments persist for several days thereafter. Markedly, based on the available documentation, regardless of the diverse range of MPTP doses given, its effects on swimming parameters remain uniform.

#### 3.6.2 Early and late MPTP effects on adult zebrafish physiology

In the days following administration, MPTP influenced the expression of genes and proteins linked to PD (Table 4). Within 24 h after injection, there was significant upregulation observed in genes associated with mitochondrial ETC (*chchd2*), ROS production (*htra2*), and autophagy process (*park2*). Conversely, significant downregulation was reported pertaining to gene associated with mitochondrial genome maintenance (*polg*; Sarath Babu et al., 2016), suggesting disturbance in normal mitochondrial activity. Meanwhile, no significant changes were found in the expression of markers for dopaminergic neurons (*th1* and *th2*) on Day 1 (Omar et al., 2023a), suggesting that the population of these neurons remained within normal levels.

**Table 4:** The early and late effects of MPTP on targeted gene and protein expression in adult zebrafish.

Days post-injection		Expression level			References
		Upregulated	Downregulated	Unchanged	
Gene expression					
Early effects	Day 1	*chchd2, crf, htra2, park2, sept3, sncgb, synb*	*polg*	*cal, dnm2a, eef2b, eif4g1, gigyfp1, hspa4l, khdrbs1a,* m*ao-b, lrrk2, park7, pink1, sncga, syn2b*	Sarath Babu et al. (2016)
			*dat*		Selvaraj et al. (2019)
				*th1, th2, dat*	Omar et al. (2023a)
	Day 2	*fis1, pink1, park2, hmgb1, tlr4, nfkb*			Razali et al. (2023)
	Day 3		*th1*	*th2, dat*	Omar et al. (2023a)
			*th1, bdnf, ntf3*	*th2, dat*	Omar et al. (2023b)
	Day 5		*th1*	*th2, dat*	Omar et al. (2023a)
			*th1*	*th2, dat, bdnf, ntf3*	Omar et al. (2023b)
Late effects	Day 10	*th2*		*th1, dat*	Omar et al. (2023a)
		*th2*	*bdnf*	*th1, dat, ntf3*	Omar et al. (2023b)
	Day 30		*th1*	*th2, dat*	Omar et al. (2023a)
Protein expression					
Early effects	Day 1	PARK8		TH, SNCGA/B, DJ1	Sarath Babu et al. (2016)
		CASP3		GST, DA	Omar et al. (2023a)
	Day 3			CASP3, GST, DA	
		DA		BDNF, GST, NT3, CASP3	Omar et al. (2023b)
	Day 5	CASP3		GST, DA	Omar et al. (2023a)
		CASP3		DA, BDNF, GST, NT3	Omar et al. (2023b)
Late effects	Day 10	GST		CASP3, DA	Omar et al. (2023a)
		GST		DA, BDNF, NT3, CASP3	Omar et al. (2023b)
	Day 30	CASP3, GST		DA	Omar et al. (2023a)
Neurotransmitter level					
Early effects	Day 1		DA		Selvaraj et al. (2019)
	Day 3				
	Day 5		DA, DOPAC, HVA		Nellore et al. (2013)

^*^Empty brackets: no data available.

Regarding the *dat* gene, while Selvaraj et al. (2019) noted downregulation on Day 1, this was not corroborated by Omar et al. (2023a), despite their similar experimental conditions and MPTP dose administered. One possible explanation for this discrepancy could be the potential influence of the circadian clock on dopamine levels through the circadian rhythm-dopamine regulatory network. The circadian rhythm coordinates daily fluctuations in gene expression, regulating various physiological functions, including dopamine-associated genes (Huang et al., 2015; Sacksteder and Kimmey, 2022). Hence, it is conceivable that the discrepancies observed between Selvaraj et al. (2019) and Omar et al. (2023a) result from the circadian influence on the *dat* gene. Possibly, their observations were conducted at different times of the day, during which the circadian clock might have influenced *dat* levels differently. Nevertheless, deeper understanding of the interconnected relationships among MPTP, the circadian clock, and the dopamine system is needed to prove this hypothesis.

Regarding protein expression, PARK8 (also known as LRRK2) and caspase-3 exhibited upregulation on Day 1. PARK8, implicated in modulating inflammatory responses and the recruitment of microglia in PD (Rui et al., 2018), tends to heighten its activity during periods of oxidative stress and endolysosomal dysfunction (Rocha et al., 2022). Conversely, caspase-3 acts as a potent mediator in neuronal apoptosis, where increased activation of this protein can initiate programmed cell death and inflammation (Imbriani et al., 2019). Markedly, there were no significant alterations in the expressions of TH and DA (Sarath Babu et al., 2016; Omar et al., 2023a). This aligns with the earlier mentioned levels of *th1* and *th2* genes, suggesting that within 24 h, MPTP may have initiated mitochondrial damage and immune cell responses, while dopaminergic neurodegeneration either has not started or remains within a manageable threshold.

Two days after injection, Razali et al. (2023) reported increased expression of three genes associated with mitochondrial dysfunction; *fis1*, *pink1*, *park2*, indicating ongoing mitochondrial damage even 48 h after MPTP administration. Besides, upregulation of neuroinflammatory genes (*hmgb1*, *tlr4*, *nfkb*) suggested an inflammatory response triggered by MPTP-induced mitochondrial damage. The HMGB1 protein, encoded by the *hmgb1* gene, is known to promote autophagy and apoptosis for mitochondrial quality control (Qi et al., 2015; Tang et al., 2011). By Day 3, Omar et al. (2023a) and Omar et al. (2023b) reported significant downregulation of *th1*, a trend seen persisting on Day 5. This temporal sequence suggests that mitochondrial damage and inflammatory response occur prior to the degeneration of dopaminergic neurons. These collective findings highlight the early effects of MPTP administration on mitochondrial activity, which appear to precede the degeneration of dopaminergic neurons, suggesting a probable link between mitochondrial dysfunction and subsequent death of dopaminergic neurons in the context of adult zebrafish PD induced by MPTP.

There was a recovery trend observed in *th1* expression by Day 10 post-injection (Omar et al., 2023a; Omar et al., 2023b), although it remained downregulated on Day 30 (Omar et al., 2023a). Interestingly, on Day 10, there was a notable upregulation of the *th2* gene (Omar et al., 2023a; Omar et al., 2023b). This increase in *th2* expression might be linked to the compensatory mechanism within the dopaminergic pathway to compensate for the loss of dopamine. In zebrafish, *th1* and *th2* are paralogous genes and are responsible for dopamine synthesis (Yamamoto et al., 2010). Notably, *th2* is more localized within the diencephalic region of the brain compared to *th1* (Filippi et al., 2010). According to Omar et al. (2023a) and Omar et al. (2023b), the expression of glutathione s-transferase (GST) protein increased from Day 10 onward, indicating a delayed reaction of this protein to MPTP activity. GST plays a pivotal role in various brain processes, including detoxifying xenobiotic molecules and stimulating microglial activation during neuroinflammation (Matoba et al., 2023). This suggests that after Day 10, the recovery process might take place to counter the effects of MPTP. Nonetheless, further data is required to comprehensively evaluate the delayed impact of MPTP on gene and protein expression in zebrafish for a conclusive assessment.

Regarding dopamine neurotransmitters, HPLC analysis revealed a 35% reduction in dopamine levels within 24 h after MPTP injection (Selvaraj et al., 2019). By Day 5, dopamine levels remained significantly low, alongside its primary metabolites, DOPAC (3,4-dihydroxyphenylacetic acid) and HVA (homovanillic acid; Nellore et al., 2013). Dopamine naturally undergoes a metabolic process within the brain, transforming into its less active metabolites, DOPAC and HVA (Masato et al., 2019; Winner et al., 2017). The decline observed in both dopamine and its metabolites in MPTP-induced zebrafish suggests a detrimental impact on dopamine metabolism, likely disrupting the normal breakdown process of dopamine following exposure to MPTP.

#### 3.6.3 Other MPTP effects on adult zebrafish

Immunohistochemistry (IHC) analysis using antibodies against tyrosine hydroxylase (TH) revealed no differences in the count of TH+ neurons between MPTP-induced zebrafish and control groups on Day 1 (Bretaud et al., 2004—whole brain; Sarath Babu et al., 2016—optic tectum region). This aligns with the gene and protein expression data that detected no alterations in TH gene and protein levels on Day 1. The immunohistology finding further supports the inference that the degeneration of dopaminergic neurons might not have started or reached a significant level within the initial 24 h post-MPTP administration. Nonetheless, Omar et al. (2023a) and Omar et al. (2023b) documented a substantial decrease in TH+ neuron counts across multiple brain regions from Day 1 to Day 30. Notably, on Day 1, there was a reduction in the number of dopaminergic neurons specifically in the preoptic region of MPTP-induced zebrafish compared to the controls. The discrepancy between Omar et al. (2023a) and the other two studies could be attributed to the distinct regions analyzed, acknowledging that different brain areas have varying populations of dopaminergic neurons. Markedly, on Days 3, 5, and 10, there was a notable decrease observed in TH+ neurons within the zebrafish posterior tuberculum (Omar et al., 2023a; Omar et al., 2023b), a region recognized for its functional similarities to the human substantia nigra. This observation further reinforces the notion that MPTP induces nigral dopaminergic neurodegeneration like those seen in PD.

Additionally, there was an immediate appearance of darkened skin pigmentation that persisted until Day 3 (Bretaud et al., 2004). It is plausible that the hyperpigmentation observed in MPTP-induced zebrafish is a result of direct or indirect effect of MPTP on melanin synthesis in dopaminergic neurons, given that neuromelanin-containing dopaminergic neurons in the SNpc are particularly susceptible in PD (Krainc et al., 2023). Apart from that, there were no significant alterations in body weight observed between Day 1 and Day 4, indicating that the influence of MPTP on appetite, particularly in the initial stages, seems negligible (Razali et al., 2022).

Bretaud et al. (2004) examined the impact of MPTP on cognition, specifically focusing on associative learning and spatial working memory. Their results showed that adult zebrafish treated with 200 μg/g MPTP displayed noticeable changes in behavior compared to the control fish. These changes included a loss in cognitive abilities like what is observed in the advanced stages of PD. Indeed, in addition to motor symptoms, PD is also linked to non-motor symptoms, including cognitive impairment. Dysfunction in the frontal cortex, which includes executive function and attention, usually occurs in the early stages, while cognitive functions related to the posterior cortex, such as memory and visuospatial abilities, tend to decline in the later stages (Sonne et al., 2022). The research conducted by Bretaud et al. (2004) has demonstrated the presence of cognitive impairment in a zebrafish PD model. This finding suggests that it is feasible and justifiable to further investigate the assessment of non-motor symptoms in zebrafish models of PD.

## 4 Discussion

The primary role of the nigrostriatal dopaminergic pathway is to regulate voluntary movement via basal ganglia motor circuits (Sonne et al., 2022). In PD, this pathway is the most severely affected, contributing significantly to the movement impairment commonly observed in PD patients. The mechanism of action of MPTP neurotoxin mimics PD physiology, wherein it triggers mitochondrial damage, that leads to detrimental cascades resulting in dopaminergic neuronal insults (Alam et al., 2017). This characteristic allows MPTP to be used to model PD in animals, including zebrafish. Investigating the immediate, early, and late effects of MPTP on adult zebrafish is pivotal for understanding the dynamic nature of its toxicity and comprehensively elucidating the progression of its pathological events.

Like any animal model, when it comes to modeling MPTP-induced PD in zebrafish, several critical factors, like zebrafish traits (like weight, age, and strain), the method of drug administration, and the dosage and frequency of drug exposure, are essential. For modeling PD in adult zebrafish via single (one-time) MPTP administration using i/p injection, studies typically involve both male and female zebrafish aged between 4 and 6 months old from the WT strain. MPTP dosages range between 20 μg/g (lowest dose) to 225 μg/g (highest dose), avoiding doses above 292 μg/g (lethal dose), and assessments are predominantly conducted 1 day after injection.

**Figure 5:**
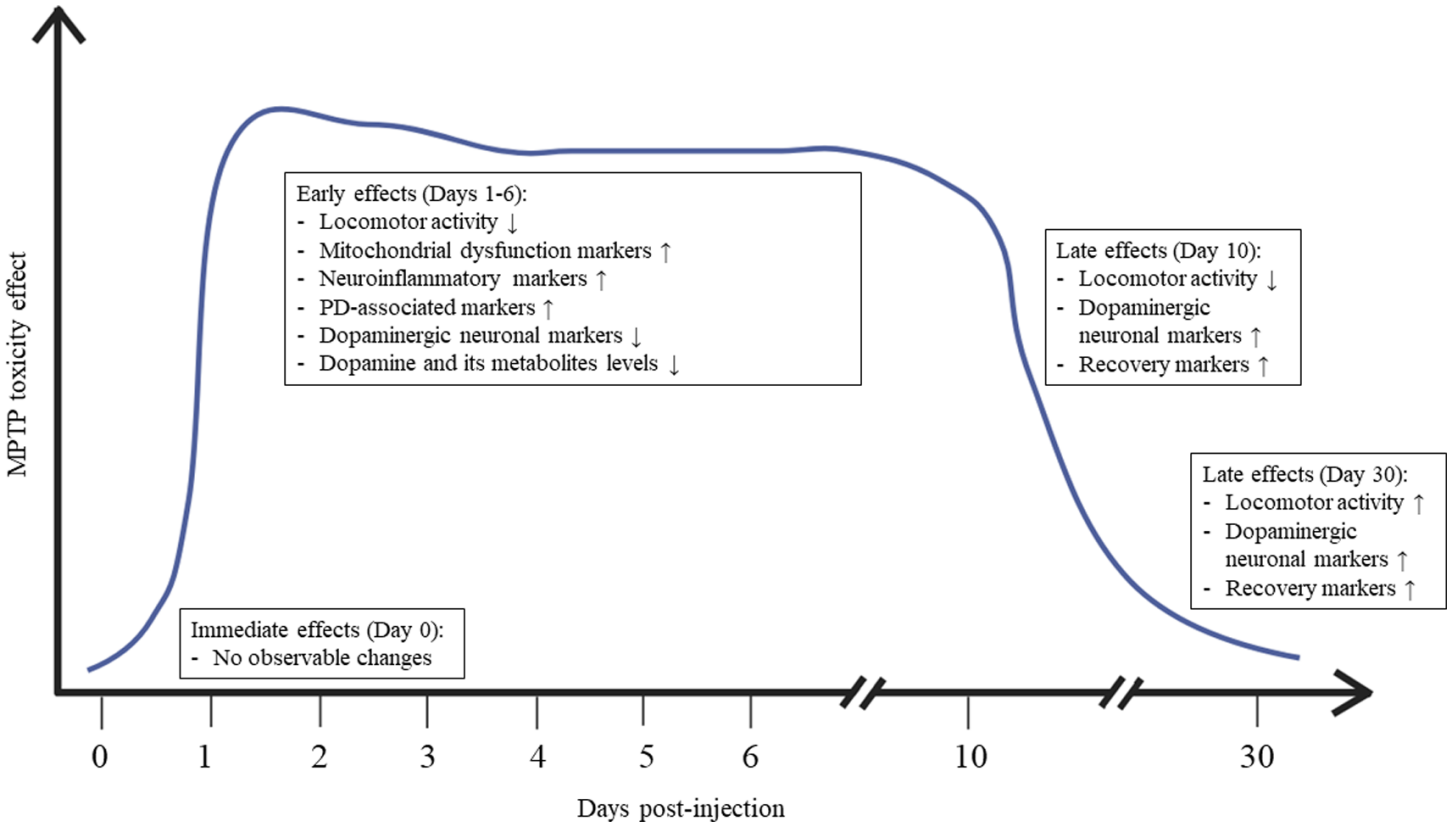
Summary of the dynamic changes in behavior and physiology of adult zebrafish following MPTP administration.

These criteria, combined with the experimental setup, induce alterations in swimming behavior and physiological insults, resulting notably in substantial locomotor deficits and changes in the expression of various mitochondria- and PD-related genes and proteins within 24 h post-injection. Markedly, signs of mitochondrial damage become apparent before the death of dopaminergic neurons, signifying that mitochondrial dysfunction occur before neuronal loss in the case of zebrafish lesioned by MPTP toxicity. Starting from the 10th day after MPTP administration, noticeable signs of improvement begin to emerge. These are indicated by heightened GST activation and a near-return to normal levels of locomotor activity. Figure 5 depicts the dynamic changes in behavior and physiology in the adult zebrafish model of PD induced by a single intraperitoneal injection of MPTP.

As discussed in earlier sections, employing adult zebrafish as a model for PD provides a more accurate representation of the typical onset stage of the disease, considering PD is often associated with aging and primarily affects the elderly. Nevertheless, several studies investigating MPTP-induced pathology have utilized zebrafish embryos and larvae. In general, both juveniles and adults exhibit a comparable response to MPTP, with disrupted activity of mitochondrial complex I and elevated expression of PD-associated genes following MPTP administration (Cansız et al., 2022; Díaz-Casado et al., 2016). However, careful consideration is needed when comparing findings between juveniles and adult zebrafish, as differences in their developmental stages and brain maturity levels may lead to distinct susceptibilities and degrees of response to MPTP toxicity. For instance, a dosage of 200 μg/g of MPTP, considered acceptable for adult zebrafish, might be excessively high for juveniles. Additionally, delivering MPTP via i/p injection may not be the most suitable method for juveniles due to their extremely small sizes and the absence of a well-defined intraperitoneal cavity, although not entirely impossible (see Saito et al., 2020). Besides, the ongoing neural development and organogenesis in juveniles contribute to a less stable and less predictable metabolism towards MPTP compared to adults (Wang et al., 2023).

## 5 Translational potential of the adult zebrafish PD model to human applications

In PD research utilizing the adult zebrafish model, the primary objective often revolves around investigating the effects of dopaminergic system dysfunction. Typically, researchers analyze zebrafish swimming behavior to elucidate locomotor activity, explore mechanisms related to neurodegeneration and neuroprotection in the context of PD, and conduct drug screening analyses and potential therapeutic development to ameliorate the dysfunction. Like other animal and pre-clinical studies, the direct translation of findings from zebrafish research to human applications presents challenges, primarily due to the substantial complexity difference between zebrafish and humans. Nonetheless, such translation is not impossible, as the zebrafish model offers several advantages.

Zebrafish share more than 70% similarity with the human genome, and over 80% of their genes are orthologous to those involved in human diseases (Benchoula et al., 2019). This substantial similarity facilitates the translation of fundamental molecular processes and disease-related mechanisms observed in zebrafish to be applicable to humans. Consequently, the zebrafish PD models, both neurotoxin-induced and genetic models, have played a big role in enhancing our understanding of the functions of genes and molecular pathways implicated in PD (Chia et al., 2022). Selecting the right type of zebrafish is crucial to ensure that the research findings are appropriately applicable. In our context, opting for adult zebrafish from the WT strain with mixed genders reflects a diverse and typical population, like the general human population. While PD may be more common in males, it can affect individuals across genders, making this choice more representative of the overall susceptibility in the human population.

A crucial factor in modeling PD is ensuring that the dopaminergic system in the chosen model organism closely resembles that of humans. Zebrafish has a well-identified dopaminergic system, with its development completed by the fourth day after fertilization (Wasel and Freeman, 2020). Additionally, specific regions in the zebrafish brain—the ventral diencephalon and sub-pallium—have been acknowledged as functionally equivalent to the human substantia nigra and striatum, respectively (Du et al., 2016). These regions are among the earliest areas affected in PD. The well-characterized dopaminergic system in zebrafish makes it a suitable model for investigating dysfunction within this system. In our context, the administration of MPTP in adult zebrafish results in mitochondrial dysfunction, mirroring the pathology observed in human PD. Understanding the pharmacokinetics of MPTP allows for a more profound exploration of the molecular mechanisms underlying mitochondrial dysfunction, offering insights into the disruption of mitochondrial activity in the development of PD. Given the high energy demands of dopaminergic neurons (Gao et al., 2022), the clear implication of mitochondrial dysfunction in both idiopathic and genetic PD emphasizes its significance in understanding the etiology of PD.

Moreover, the zebrafish has proven to be a valuable model for studying locomotor dysfunction in PD. While it is acknowledged that zebrafish behavior and movement are less complex than those of humans, the impaired movement observed in zebrafish induced with PD closely mirrors the motor symptoms seen in human PD. In our context, we noted a decrease in zebrafish locomotor activity following the administration of MPTP. This observation resembles the slowness of movement seen in PD patients, or more commonly known as bradykinesia. In addition to locomotor dysfunction, the high similarity of zebrafish genes to humans allows for the investigation of PD pathology at the molecular level. Several genes associated with PD and mitochondrial dysfunction show upregulation in MPTP-induced zebrafish, as shown in previous sections. The ability of the zebrafish model to mimic PD symptoms and exhibit pathology like humans offers a valuable platform for testing potential mechanisms to prevent PD and apply those findings to humans.

## 6 Limitations and future recommendations

While this review has systematically examined the behavior and physiology of the MPTP-induced zebrafish model of PD, it does have some limitations. A major constraint of this review is the relatively restricted number of relevant studies available, which limits the potential for thorough comparisons among their conclusions. The scarcity of studies may be attributed to the strict inclusion criteria employed, which exclusively target a single neurotoxin (MPTP) and a specific method of delivery (i/p injection). To overcome this constraint, the search method can be enhanced by using additional platforms such as the Web of Science and SCOPUS. These platforms may include papers that are not listed in PubMed and Google Scholar. Furthermore, the inclusion of non-original research materials such as conference proceedings and dissertations might enhance the depth of the findings.

Furthermore, to reduce the number of factors that could affect the results, we specifically chose studies that administered MPTP through i/p injection. We deliberately omitted studies that utilized alternative routes of administration such as intramuscular injection (Anichtchik et al., 2004), cerebroventricular injection (Kalyn and Ekker, 2021), and immersion (Anichtchik et al., 2004; Kalyn and Ekker, 2021; Saszik and Smith, 2018). The rationale for this is that various methods of delivery can influence the onset at which MPTP takes effect, the dosages, and the severity of the observed symptoms. For example, the rate at which MPTP is absorbed, distributed, metabolized, and excreted may differ depending on whether it is administered via immersion or cerebroventricular injection. While our inclusion criteria effectively reduced the diversity in approach, it also resulted in a smaller number of relevant studies being included in this systematic review. To address this limitation, the selection criteria could be expanded to encompass other methods of administration. This would enable a more comprehensive comparison of results and emphasize the impact of variations caused by various delivery methods.

A drawback of utilizing zebrafish as a model for evaluating PD symptoms is the limited number of validated assessments. While mouse models have been extensively studied and are widely accepted, zebrafish models are still in the process of being developed. Consequently, there are only a few types of assessment methods that are specifically designed to examine symptoms linked to PD, especially the non-motor symptoms. Additionally, only a few studies have conducted these assessments, limiting the ability to make comprehensive comparisons. The rotenone-induced adult zebrafish PD model has been reported to show PD-associated non-motor symptoms such as anxiety, depression, and olfactory impairment (Wang et al., 2017). Conducting similar assessments on the MPTP-induced model could provide valuable insights into the effects of MPTP toxicity on non-motor symptoms, as well as highlight similarities and differences compared to other toxins. Additionally, incorporating these assessments can enhance the adult zebrafish model of PD and improve the sensitivity of observations. Assessing cognitive impairments linked to PD, such as anxiety, memory and visuospatial deficits, could significantly enhance our comprehension of how MPTP impacts the zebrafish PD model.

Lastly, there is a notable lack of studies investigating the long-term effects of MPTP toxicity on adult zebrafish. Among the studies selected for this review, only one extended observation up to 30 days post-administration. This scarcity of long-term studies limits our understanding of the progressive nature of the disease. Long-term observations in the zebrafish PD model are particularly valuable as they can provide insights into the zebrafish regenerative abilities concerning the affected dopaminergic system. Zebrafish are well known for their capacity to regenerate neurons (Zambusi and Ninkovic, 2020), and findings from such studies could potentially inform strategies for protecting and restoring dopaminergic neurons in humans. However, conducting long-term observations is challenging due to the transient nature of MPTP effects in zebrafish. To address this, multiple administrations over a designated period might be necessary to establish more enduring effects of MPTP.

Despite these challenges, this review contributes significantly to our understanding of how this neurotoxin can be used in developing zebrafish PD models. It provides valuable insights into the effects of MPTP in these models and lays the groundwork for establishing alternative zebrafish PD models, such as sub-acute, sub-chronic, and chronic models.

## 7 Conclusion

The administration of MPTP neurotoxin effectively induces PD in adult zebrafish. Through a single i/p injection, noticeable parkinsonian symptoms appear 1 day after administration and persist for more than 1 week. Mitochondrial dysfunction precedes dopaminergic neurodegeneration within this experimental regime. Our review provides a framework for future studies, highlighting the optimal timing for assessing MPTP effects, expected behavioral and physiological alterations, and the most appropriate window for planning therapeutic interventions to counter MPTP-induced neurotoxicity.

## Data Availability

The raw data supporting the conclusions of this article will be made available by the authors, without undue reservation.
